# Integration of computed tomography and intravascular ultrasound for optimal management of chronic total occlusions with intramyocardial bridge: a case report

**DOI:** 10.1093/ehjcr/ytaf064

**Published:** 2025-02-06

**Authors:** Giuseppe Panuccio, Salvatore De Rosa, Daniele Torella, Ulf Landmesser, Youssef S Abdelwahed

**Affiliations:** Department of Experimental and Clinical Medicine, Magna Graecia University, Viale Europa, 88100 Catanzaro, Italy; Department of Cardiology, Angiology and Intensive Care Medicine, Deutsches Herzzentrum der Charité, Hindenburgdamm 30, 12203 Berlin, Germany; Department of Medical and Surgical Sciences, Magna Graecia University, Viale Europa, 88100 Catanzaro, Italy; Department of Experimental and Clinical Medicine, Magna Graecia University, Viale Europa, 88100 Catanzaro, Italy; Department of Cardiology, Angiology and Intensive Care Medicine, Deutsches Herzzentrum der Charité, Hindenburgdamm 30, 12203 Berlin, Germany; DZHK (German Centre for Cardiovascular Research), Berlin, Germany; Berlin Institute of Health (BIH), Berlin, Germany; Department of Cardiology, Angiology and Intensive Care Medicine, Deutsches Herzzentrum der Charité, Hindenburgdamm 30, 12203 Berlin, Germany; DZHK (German Centre for Cardiovascular Research), Berlin, Germany

## Summary

Chronic total occlusions (CTOs) are a challenging scenario in coronary artery disease. Coronary computed tomography (CT) has becoming increasingly valuable in CTO-percutaneous coronary intervention (PCI), by also allowing pre-procedural identification of intramyocardial bridges (IMBs), which impact long-term outcomes in CTOs.^1^

## Case description

**Figure 1:**
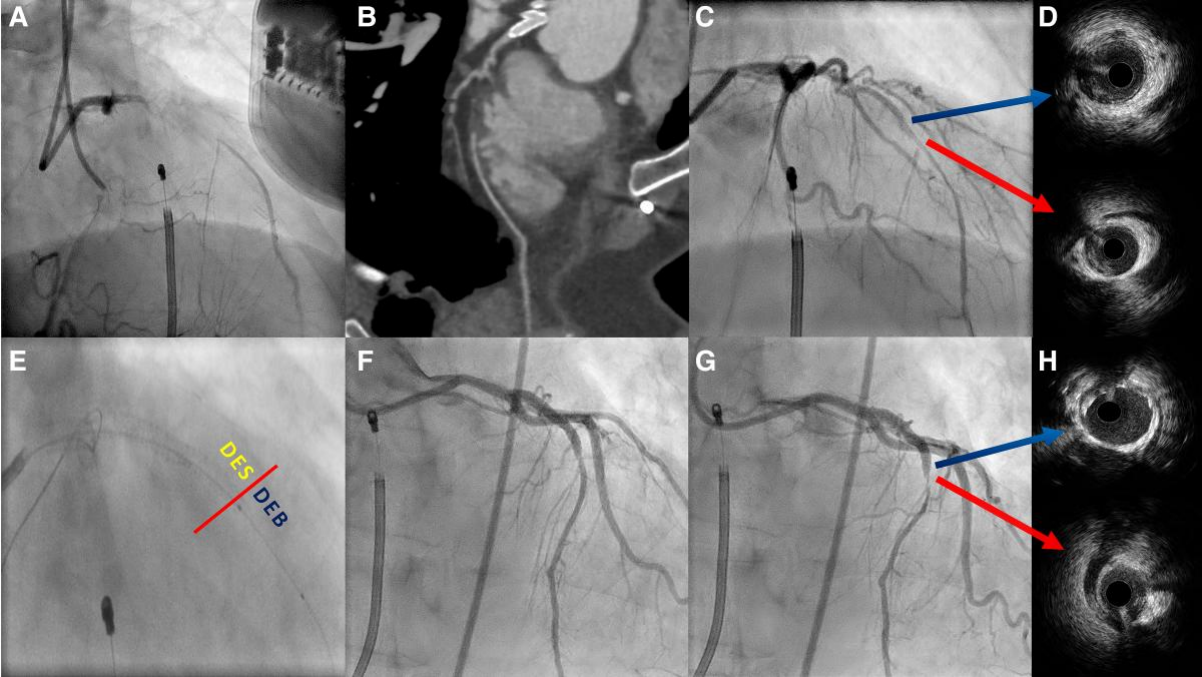
(*A*) Baseline angiography showing the occluded LAD segment; (*B*) CT evidence of intramyocardial bridge within the CTO lesion; (*C*) coronary flow restore after balloon; (*D*) IVUS of the CTO area showing the boundary between IMB (with the typical ‘half-moon’ sign) and IMB-free area; (*E*) boundary between DES-treated area (without IMB) and DEB-treated area (within IMB course); (*F* and *G*) final result; (*H*) IVUS confirming IMB-area spared from stent implantation.

An 80-year-old male with refractory angina (CCS III) despite optimal medical therapy and prior coronary artery bypass grafting presented with an occluded graft and a mid-left descending artery (LAD) CTO (*Figure 1A*; Supplementary material online, *Video S1*), with extensive collateral circulation and stress echocardiogram showing anterior wall viability. Pre-procedural CT revealed an IMB compressing the distal CTO segment, complicating its management (*Figure 1B*). Computed tomography detects IMBs by identifying systolic luminal narrowing (milking-effect), helping assess their haemodynamic significance. Computed tomography findings allowed a pre-procedural planning of a conservative approach for the IMB-affected segment. The lesion was crossed by a high-penetration guidewire (Gaia 1™, Asahi-Intecc, Japan), and non-compliant balloon pre-dilation restored the flow (*Figure 1C*). Intravascular ultrasound (IVUS), according to plaque-burden and minimal-lumen-area, accurately identified an optimal stent-landing zone, also sparing the IMB-affected segment (detected with its typical half-moon sign, *Figure 1D*), which was treated with a drug-eluting-balloon (DEB, *Figure 1E*). This strategy avoided potential IMB-related complications such as in-stent restenosis or fracture.^2^ The procedure achieved excellent results (*Figure 1F* and *G*), with IVUS confirming IMB-sparing from stenting (*Figure 1H*), and with no residual angina reported (along with beta-blocker treatment).

Computed tomography and IVUS integration allowed precise planning and optimization of CTO-PCI, highlighting the role of coronary imaging in managing complex CTO cases involving IMBs. Conservatively sparing IMB-affected segments with DEB prevented stent-related complications, as mechanical stress caused by IMBs can lead to stent failure.^3^ Since long-term data are still limited, this case shows this approach’s feasibility and safety, thanks to coronary imaging support.

Integrated precision-medicine approaches with coronary CT and IVUS are crucial in identifying IMBs and guiding CTO-PCI management, minimizing harmful stenting and improving outcomes.

## Supplementary Material

ytaf064_Supplementary_Data

## Data Availability

The data underlying this article will be shared on reasonable request to the corresponding author.

## Supplementary material

**Consent:** Written informed consent was obtained from the patient to publish this report in accordance with the journal’s patient consent policy.

**Funding:** None declared.
