# Supplementary material for: Integration of computed tomography and intravascular ultrasound for optimal management of chronic total occlusions with intramyocardial bridge: a case report
Source: Eur Heart J Case Rep. 2025 Feb 6;9(2):ytaf064. doi: 10.1093/ehjcr/ytaf064 (PMC11851276; doi:10.1093/ehjcr/ytaf064)
Supplement: ytaf064_Supplementary_Data [file ytaf064_supplementary_data.pptx]

## Slide 1
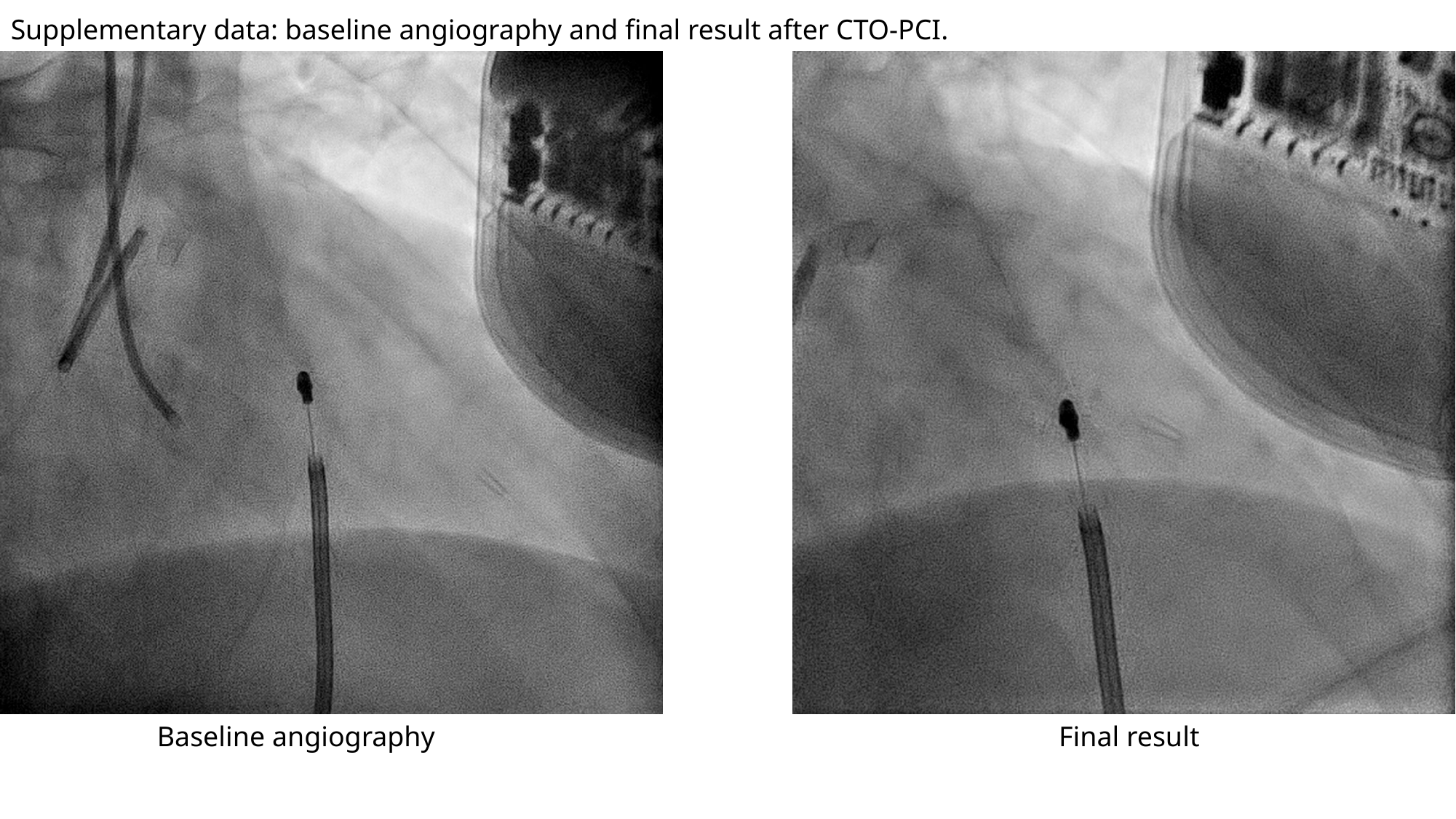

Supplementary data: baseline angiography and final result after CTO-PCI.
Baseline angiography
Final result
